# The Influence of Fillers on the Reinforcement Capabilities of Polypropylene Based Mono-Material and Core-Shell Fibers in Concrete, a Comparison

**DOI:** 10.3390/polym17131781

**Published:** 2025-06-27

**Authors:** Jonas Herz, Dirk Muscat, Nicole Strübbe

**Affiliations:** Faculty of Engineering Sciences, Rosenheim Technical University of Applied Sciences, Hochschulstraße 1, 83024 Rosenheim, Germany

**Keywords:** fibers, extrusion, coextrusion, polymer drawing, polypropylene, calcium carbonate, bentonite, core-shell, mono-material, concrete

## Abstract

Noncorrosive concrete reinforcement, such as polymer fibers, is needed to overcome the current issues caused by corroded steel reinforcements. Fibers made of polypropylene show a low bonding behavior in concrete. Fillers can help to overcome this issue but often lead to reduced mechanical properties. Core-shell fibers, which split the mechanical properties and the bonding behavior between the core and the shell component, could be a solution. This study investigates mono-material and core-shell fibers produced with calcium carbonate and bentonite fillers and compares their behavior in tensile tests, density measurements, contact angle measurements, topography measurements, single fiber pull-out tests, reflected light microscopy, and thermogravimetric analysis. The fillers caused an increased drawability, resulting in higher mechanical properties. Further, in the core-shell fibers, the calcium carbonate increased the surface roughness, which led to a better anchoring of the fiber in concrete, which was also visible in the deformation during pull-out observed in reflected light microscopy pictures. The thermogravimetric analysis showed a delay in onset of degradation for fibers containing bentonite.

## 1. Introduction

Concrete in its different variations is a material that is used worldwide in huge quantities [1]. Besides many advantages like high compressive strength, availability of raw materials, and design/form freedom, concrete has a low tensile strength [2,3,4,5,6]. To overcome this issue, during the 20th century, steel became the reinforcement material to enhance the tensile resistance of concrete [7]. However, the use of steel reinforcements also shows disadvantages like their corrosive behavior. Corrosion of steel in concrete can be induced by either chloride ions or by carbonation and causes first rust formation and later cracking and spalling of the protective concrete cover above the reinforcement [6,8,9]. If cracking and spalling occur, the structure’s fully effective service life ends [8,9,10].

Finding alternative reinforcement possibilities for concrete has received a lot of research attention, and different approaches have been investigated. A number of scholars conducted research on non-corrosive materials like fiber-reinforced plastic bars [11,12], textile fabrics made from glass [13,14] or carbon [15,16] and different types of fibers such as stainless steel [17], natural [18,19], glass [20,21], carbon [22], and polymer [20,21,23] fibers as concrete reinforcement. To achieve a reinforcement effect, fibers have to fulfill two major requirements. The fibers need high mechanical properties and a good anchoring in the concrete matrix [24]. In the case of polymer fibers, the polymer from which the fiber is produced has a high influence on the later fiber properties, e.g., a fiber produced from polypropylene (PP) lacks a good fiber matrix interaction due to its hydrophobic surface [25], or a fiber produced from polyethylene terephtalate is partially degraded in the alkaline environment [26].

However, there are different possibilities to influence the mechanical and anchoring properties of polymers and polymer fibers. The mechanical properties of polymers can be enhanced by generating high molecular orientation using a stretching or drawing process [27]. Multiple methods were described to attain molecular orientation by polymer drawing. A basic method is cold drawing, which was first described by Carothers and Hill [28], where temperatures below the melting temperature of the polymer are used [29]. A simple procedure of a discontinuous cold drawing process is to clamp a polymer in a tensile testing machine using the testing speed to apply a stress that causes the molecular orientation until a predefined draw ratio (DR) or drawing time is reached, like described by Wills et al. [30] or Coppola et al. [31]. The disadvantage of this method is that just one draw ratio can be produced per clamped sample. Alternatively, a continuous drawing method using a setup with two rolls or godets with different speeds and a heating system between them can be applied to achieve molecular orientation in different DRs without further material handling. These drawing units can be either placed inline in an extrusion process [32] or offline to draw separately produced strands, which enables the opportunity to also use more than one drawing step [33,34,35]. Although polymer drawing can happen also at room temperature, often an enhanced temperature is used for polymer drawing. The literature shows different heating options for drawing processes, like air-heated furnaces [32,33] or baths with heated liquids like glycerol [36] or water [37].

Another possibility to improve the mechanical properties of a polymer is the addition of fillers [27,38]. But the addition of fillers combined with a drawing process will result in void formation if the adhesion between filler particle and polymer matrix is weak [39,40]. Further, large filler particles or poorly dispersed agglomerates lead to early crack formation and filament breaking [40,41]. To achieve a better filler dispersion and filler matrix adhesion, a coupling agent can be used [42]. Sigrüner [43] observes a decrease in mechanical properties of drawn PP containing macro sized fillers with and without a coupling agent. In the case of nano-sized fillers with a coupling agent, an enhancement was possible. On the other hand, fillers can be used to enhance the bonding between polymer fiber and concrete, as presented by single fiber pull-out tests (SFPTs) in a previous study [35].

There exist different approaches to increase the bonding between polymer fibers and concrete matrix, e.g., enhancing the contact surface between fiber and concrete by triloba [25], or x-shaped [44] fiber cross sections. Another possibility to enhance the anchoring of polymer fibers in concrete is the use of surface and structure modifications like crimped [45,46] or embossed [45,46,47] structures, hooked or enlarged fiber ends [48], or fibrillation and twisting [49]. This surface structuring creates undercuts, which improves the transmission of forces between fiber and concrete [50]. Fiber roughness acts in the same way and is accordingly important for anchoring polymer fibers in a concrete matrix [25,32]. It is believed that the earlier mentioned enhanced bonding between filler-containing fibers and concrete is due to increased surface roughness caused by the filler [35].

Multiple types of polymers have been investigated by different authors regarding their suitability for concrete reinforcement fibers. The analyzed fiber materials range from standard polymers like polyethylene [51], and polypropylene [52] to technical ones, e.g., polyamide [53] or polyethylene terephtalate [54] and high-performance plastics like polyetheretherketone [32]. The advantages of PP as a fiber material are a low price [25], its chemical inertness, which makes it stable even in highly corrosive environments [55] and its low density. The density of PP fibers lies in the range of 0.895 to 0.920 g/cm^3^ [50]. Other fiber reinforcement materials for concrete show much higher densities, e.g., steel (7.85 g/cm^3^ [56]), glass (2.7 g/cm^3^ [57]), or carbon (1.74–1.80 g/cm^3^ [58]). However, the main disadvantage of PP is the low bonding to concrete due to its chemical inertness [25]. To overcome this issue, the addition of a filler material can be considered. But, as described earlier, the filler can cause decreased mechanical properties. Current research seems to indicate that the use of a bicomponent fiber with a core-shell (c-s) structure where the core supplies the mechanical properties while the shell provides the bond to the concrete matrix could be a solution to avoid reduced fiber mechanics while maintaining the anchoring effect [59,60].

Until now, different approaches with c-s polymer fibers as concrete reinforcement have been investigated. Popa et al. [59,61] tested fibers with a PP core and compounds of PP with aluminum oxide or calcium carbonate (CC) as shell materials and retrieved promising results, like enhanced mechanical properties and surface roughness. Other attempts explored the combination of polyketone in the fibers’ core and unmodified as well as maleic anhydride-modified PP in the shell [60,62]. An investigation with neat PP in the core and PP compounded with CC and wood particles as shell material showed a reduction in mechanical properties even if the filler is only in the fibers’ shell, while a good anchoring could be reached [63]. Further, results presented by Sigrüner [43] indicate that the fillers could also be used in the fibers’ core.

Currently, there are no comparative studies between filler-containing mono-material (m-m) and core-shell fibers with fillers in either the core or shell or both of them. Accordingly, this study investigates the differences and similarities of m-m and c-s fibers produced from PP compounded with two filler materials. First, the materials are produced by compounding. Then, the fibers are produced by extrusion of m-m and coextrusion of c-s strands and a later cold drawing process. Finally, the fibers are analyzed and compared. The mechanical properties are determined using tensile tests. Further, the fiber density of the fibers is measured. The anchoring and bonding behavior is evaluated by SFPTS. Additionally, the fiber surface is investigated by contact angle and topography measurements. Pictures created by reflected light microscopy show changes of the fiber surface caused by the pull-out process, and results from thermogravimetric analysis give insight into the thermal degradation behavior of the m-m and c-s fibers.

## 2. Materials and Methods

### 2.1. Materials

**Figure 1:**
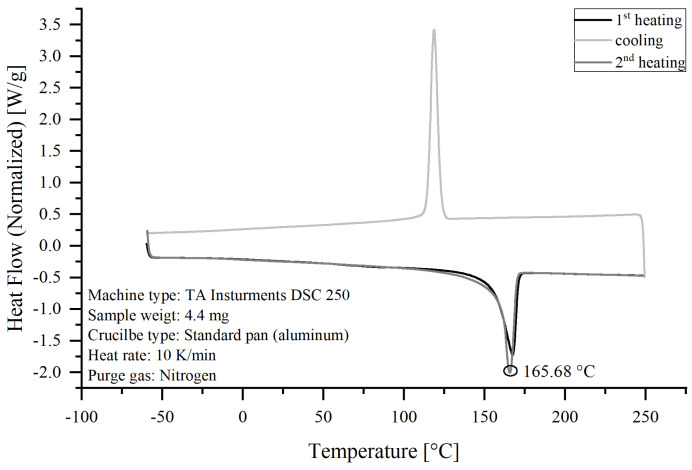
DSC analysis of the raw PP used in this study.

In this study, an isotactic PP homopolymer was used. The producers’ specifications state a melt flow rate of 1 g/10 min measured at 230 °C and 2.16 kg and a density of 0.905 g/cm^3^ at 23 °C [64]. The PP was analyzed using differential scanning calorimetry (DSC) to evaluate its melting temperature, and the PP’s melting temperature was ≈165 °C, as presented in Figure 1.

A block-like-shaped calcium carbonate (CC) and a platelet-shaped bentonite (BT) were chosen as fillers. The d_60_ of the CC is <2 µm and the d_50_ of the BT is <10 µm, as specified by the producers [65,66]. The compatibilzier used was a maleic anhydride grafted polypropylene (MAH) with a melt flow rate of 28 g/10 min at 230 °C and 2.16 kg and a density of 0.90 g/cm^3^ [67].

To study the fiber matrix bonding, the polymer fibers were embedded in concrete. The used concrete mixture consisted of the following dry additives: a CEN standard sand [68], CEM I 52.5 N Portland cement, and limestone powder. Further, as liquid additives, a polycarboxylate ether-based superplasticizer (superplasticizer 1) and a viscosity-modifying agent based on high molecular copolymers (superplasticizer 2) were added for viscosity and workability control together with water from the common tap water system. The amounts of the materials in grams and weight percent (wt.%) are presented in Table 1.

### 2.2. Processing

#### 2.2.1. Compounding

The different material combinations investigated in this study were mixed in a compounding process. Prior to compounding, all materials were predried for at least 10 h at 80 °C. The drying of polymeric components happened using dry air dryers, while the filler particles were predried in a vacuum furnace. For compounding, a Coperion (Stuttgart, Germany) ZSK MC^18^ twin-screw extruder with a length-to-diameter (l/d) ratio of 48 was used. Two different screw configurations, S1 and S2, were set up for the compounding of CC and BT. For S1, more backwards-conveying kneading blocks were used in the dispersive mixing zone compared to S2, while S2 was set up with more distributive mixing elements. On both screw configurations, also the neat PP material without fillers was processed to identify a possible influence by the compounding process. In the case of the CC compound, an MAH was added as a compatibilizer. The produced materials were named by the polymer and the used filler as indicated in Table 2. For the mixtures with fillers, 5 vol.% of CC and 2.5 vol.% of BT were added to the PP. In the case of the CC mixture, MAH was also added by 50 wt.% of the filler loading.

Table 2 shows all four materials processed on the compounder with their compositions in wt.%. The material mixtures and screw configurations used in this study were derived from a huge number of experiments that were performed in an earlier research stage (unpublished).

The polymeric materials were fed through the main hopper, while the fillers were supplied by the side feeder.

For all compounds, vacuum degassing was applied. Further, the screw speed and throughput were fixed at 300 rpm and 10 kg/h. During compounding of PP-CC-S1, a mass temperature of 211 °C, a pressure of 45 bar, and a torque of 45% were observed. The residence time of S1 measured during the production of PP-S1 was 50 s from the hopper and 45 s from the side feeder. PP-BT-S2 was compounded two times to achieve an enhanced residence time and a better deagglomeration. First a master batch with 10 vol.% was produced, which was diluted to 2.5 vol.% in a second step. During the first compounding, a mass temperature of 210 °C, a pressure of 44 bar, and a torque of 43% were measured. These values changed slightly to a mass temperature of 206 °C, a pressure of 48 bar, and a torque of 42% during the second compounding step. Also for S2, the residence times were measured while PP-S2 was produced. The residence time for S2 was 42 s from the main hopper and 35 s from the side feeder.

#### 2.2.2. Fiber Production

**Figure 2:**
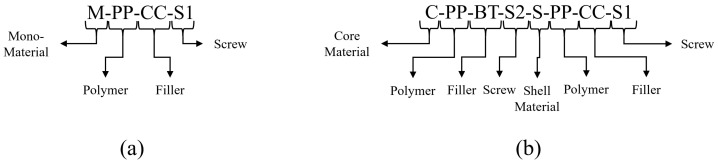
Sample code of the produced m-m (**a**) and c-s (**b**) fibers investigated in this study.

Both fiber types, the m-m as well as the c-s fibers, were produced in a two-step process. First, a strand was produced. Then, the strand was drawn to a fiber in the second step. In addition to the compounded materials, two reference fibers containing unprocessed PP were produced. The strands and fibers are labeled using the sample code explained by Figure 2.

**Figure 3:**
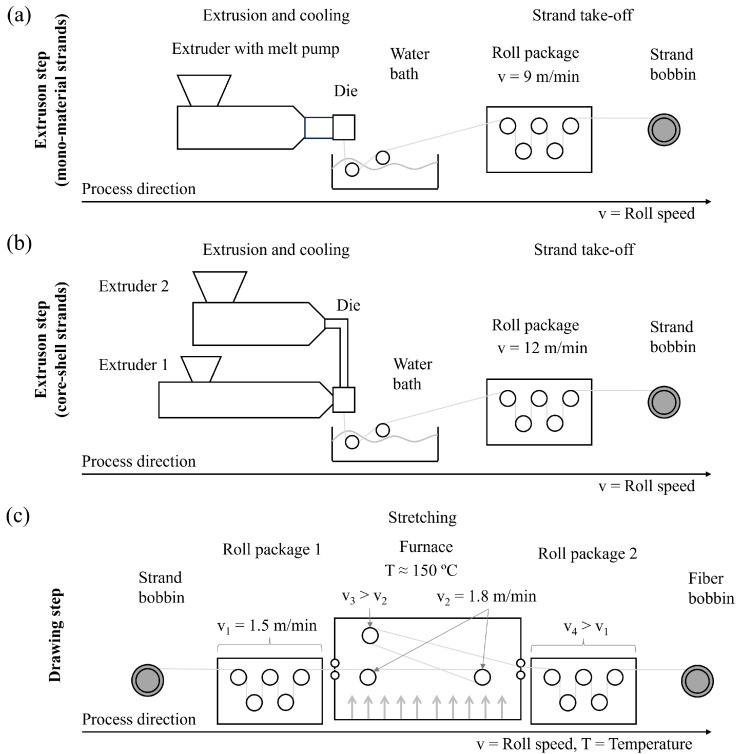
Scheme of the fiber production process: (**a**) production of mono-material strands (**b**) production of core-shell strands (**c**) drawing process that was applied on both strand types. The light grey line indicates the direction of the strands during extrusion and drawing.

The strands for the m-m fibers were produced by an extrusion process, which is shown schematically in Figure 3a. For extrusion, a HAAKE PolyDrive single-screw extruder (Thermo Electron Corporation, Waltham, MA, USA) equipped with a melt pump was used. The extruder’ screw has a diameter of 19 mm and an L/D ratio of 25. The throughput was kept constant at 1.5 kg/h by the melt pump. The other extrusion parameters can be obtained from Table 3. The produced strand was cooled in a temperature-controlled water bath at ≈58 °C, taken off by a roll package with a speed of 9 m/min, and rolled up on a bobbin.

The strands for C-S fibers were produced using coextrusion. Therefore, the Coperion double-screw extruder, which was also used for the compounding, was equipped with a moderate conveying screw configuration and a coextrusion die. The same Haake single-screw extruder that was also used for the production of m-m strand was connected to the coextrusion die via a heated hose. The strand was transferred into a water bath for tempered cooling at ≈57 °C and taken off by a roll package at 12 m/min and rolled up on a bobbin (see Figure 3b). The double-screw extruder (Extruder 1) processed the core (C) material, while the single-screw extruder (Extruder 2) supplied the shell (S) material. The coextrusion parameters are shown in Table 4. An improvement of the previously presented sample code is necessary to identify the C and S material. Therefore, C and S are put in front of the core and shell materials, e.g., C-PP-S-PP-CC-S1, where the unprocessed PP is in the fiber core and the CC compound is in the fiber shell.

The applied drawing process was the same for m-m and c-s fibers. A custom-made drawing unit was used, which is shown schematically in Figure 3c. The extruded and coextruded strands were placed in front of the drawing unit, taken up by a first roll package with a fixed speed (v_1_) of 1.5 m/min, and delivered into a furnace heated at 150 °C, which fits the recommended drawing temperature between 10 and 20 °C below the melting temperature of the used polymer, according to Dahlmann et al. [69]. There are three rolls in the furnace. The first two (v_2_) are with a speed of 1.8 m/min, just slightly faster than v_1_ to keep the strand tight on the rolls. The speed of the third roll (v_3_) and the second roll package (v_4_) is increased to apply a tension to the heated strand and to cause with that the fiber drawing. The ratios between v_2_ and v_3_ and v_3_ and v_4_ are kept approximately the same. The drawn fibers were rolled up on another bobbin. All strands were drawn until fiber failure occurred during the drawing process.

The draw ratio is defined by the speeds of the two roll packages as:

(1) $$DR=\frac{v_{4}}{v_{1}}$$

where $v_{1}$ is the speed of the roll package in front of the furnace while $v_{4}$ is the one following it.

### 2.3. Analyses

#### 2.3.1. Tensile Tests

The mechanical properties of the m-m and c-s fibers were tested using a ZwickRoell Z100 universal testing machine (ZwickRoell GmbH & Co. KG, Ulm, Germany). For the measurements, the universal testing machine was equipped with 90° pneumatic deflection grips, a 10 kN load cell, and an extensometer for measuring elongation during determination of Young’s modulus. Young’s modulus was tested at a speed of 1 mm/min between 0.05% and 0.25% elongation according to DIN EN ISO 527-1 [70]. Then the test speed was increased to 10 mm/min as recommended by DIN EN 14889-2 [71] until fiber failure to evaluate the tensile strength. The cross sectional area was determined by measuring the major and minor axis of an ellipse and calculated by [72]:

(2) $$A=a_{e}\cdot b_{e}\cdot \pi$$

whereas $a_{e}$ is the major and $b_{e}$ is the minor axis of the elliptical cross section of the fibers. Five samples were tested for each fiber, and the average of Young’s modulus and tensile strength were determined.

#### 2.3.2. Density Measurements

The density of the fibers was measured with a Mettler-Toledo MS304TS/00 scale equipped with a density kit (Mettler-Toledo, International Inc., Greifensee, Switzerland) using the archimedic principle. The test liquid was demineralized water. For every fiber and draw ratio, five samples with a weight in air of 10 ± 5 mg were taken.

#### 2.3.3. Single-Fiber Pull-Out Test

To produce the specimen for the SFPT, a single fiber was cleaned with ethanol and fixed with an embedment length of 15 mm in the center of a cubical mold with a size of 60 × 60 × 30 mm. The mold was then filled with the concrete mixture shown in Table 1, followed by vibrating the molds for degassing. During concrete curing, the molds were stored in a climate regulated chamber at 67% humidity and 20 °C. After 24 h, the specimen were demolded and stored for additional 13 days under water in the same climate chamber.

Testing took place after 14 days to measure the bonding ability of the fibers. For the SFPT, the specimen was fixed on the bottom of a ZwickRoell 1474 tensile testing machine (ZwickRoell GmbH & Co. KG, Ulm, Germany) equipped with a 500 N load cell. The fiber was clamped using a clamping jaw directly above the concrete to avoid influences by a free fiber length, as described by Zhandarov et al. [73]. The test started after a preload of 2 N was reached. The constant test speed was 2 mm/min, and the test ended with complete fiber extraction. The force was detected for the complete fiber displacement, and the maximum extraction force was used to calculate the interfacial shear strength (IFSS) by the common equation [74]:

(3) $$\tau_{IFSS}=\frac{F_{max}}{A}$$

whereas $\tau_{IFSS}$ is the IFSS, $F_{max}$ is the maximum extraction force reached during pull-out and *A* is the embedded lateral surface of the fiber. The fibers’ embedded lateral surface is calculated by:

(4) $$A=c_{f}\cdot l_{e}$$

with $c_{f}$ as the fibers’ circumference and $l_{e}$ as embedment length. and, the circumference of the elliptical fiber can be approximately calculated by [72]:

(5) $$c_{f}\approx \frac{\pi }{2}\left[3\left(a_{e}+b_{e}\right)-2\sqrt{a_{e}\cdot b_{e}}\right]$$

where $a_{e}$ and $b_{e}$ are again the major and minor axes of an ellipse.

For every fiber material five specimen at the maximum DR were tested and the average IFSS was calculated.

#### 2.3.4. Contact Angle Measurements

Contact angle measurements were carried out for all fiber materials at their maximum DR on a KRÜSS EasyDrop FM40 (Krüss GmbH, Hamburg, Germany). The fibers were cleaned using ethanol and after drying tested with distilled water and diiodomethane. Seven single small droplets were set on the fiber surface for each test liquid. The KRÜSS ADVANCE software (Version 1.6.1.0) was used to measure the contact angles of the droplets and to calculate the surface energy with the method of Owen, Wendt, Rabel, and Kaelble. The procedure also allows us to split the surface energy into a polar and a disperse part [75,76].

#### 2.3.5. Topography Measurements

The fiber surfaces were recorded by confocal laser scanning microscopy (CLSM) using a Zeiss LSM 800 (Carl Zeiss AG, Oberkochen, Germany). A C Epiplan-APOCHROMAT 50×/0.95 DIC lens was used to capture stacked images with a size of 127.78 µm × 127.78 µm and a resolution of 1228 × 1228 pixels. The distance between two layers was 0.2 µm. The resulting 3D surface images were analyzed in the Mountains 9 software (Version 9.2.10042), where the surface curvature of the fibers was eliminated and the arithmetic mean deviation of the surfaces (S_a_) was calculated. Areas, where the fiber surface structure hindered the image recording by abrubt slope changes or undercuts occurred just in very small amounts. For every fiber type, three measurements were executed.

#### 2.3.6. Reflected Light Microscopy

For evaluating the fibers before and after pull-out, reflected light microscopy (RLM) was used. For the evaluation, a Zeiss Smartzoom 5 microscope (Carl Zeiss AG, Oberkochen, Germany) was equipped with a PlanApo D 1.6×/0.1 FWD 36 mm lens. The images were taken at 200× magnification under coaxial light using a stacking routine in the software to create an extended depth-of-field for completely sharp fiber surfaces.

#### 2.3.7. Thermogravimetric Analysis

The decomposition of the fibers due to increased temperatures was analyzed by thermogravimetric analysis (TGA). The tests were executed using a TA Instruments TGA 5500 (TA Instruments, New Castle, DE, USA) with platinum pans. A single sample was tested for every fiber at their maximum DR. The samples reached weights of 10 ± 0.3 mg. All samples were heated between ≈35 °C and 650 °C with a heating rate of 20 K/min under a nitrogen atmosphere, and the mass was recorded as a function of temperature.

## 3. Results and Discussion

### 3.1. Mechanical Properties

Table 5 shows the equivalent fiber diameter, a calculated aspect ratio, and the Young’s modulus and tensile strength for all produced fibers at their maximum DR. M-PP-BT-S2 shows the highest DR of 18 as well as the highest aspect ratio and mechanical properties. The reference fibers M-PP and C-PP-S-PP, which show the lowest maximum DR of 12, also show the highest fiber diameters and the lowest mechanical properties of all fibers.

**Figure 4:**
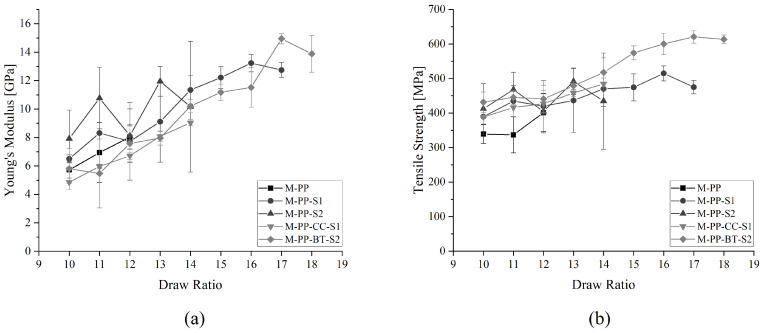
Mechanical properties of mono-material fibers at different DRs: (**a**) Young’s modulus and (**b**) tensile strength.

The mean average values of Young’s modulus and tensile strength for the different DR reached by the different m-m fibers are shown in Figure 4. The neat M-PP reference reaches the lowest DR of 12, where it also shows the highest tensile strength of 401.10 ± 54.31 MPa and Young’s modulus of 8.03 ± 0.85 GPa. The two compounded but unfilled PPs reached higher DR and also higher mechanical properties. M-PP-S1 gained a maximum DR of 17, while the highest tensile strength was 514.88 ± 21.65 MPa and Young’s modulus of 13.22 ± 0.60 GPa were reached at DR = 16. The tensile strength and Young’s modulus of M-PP-S2 were 491.72 ± 38.68 MPa and 11.94 ± 1.04 GPa at DR = 13. The highest DR reached by M-PP-S2 was 14. In the case of the m-m fibers containing fillers, M-PP-CC-S1 achieved its highest mechanical properties at its maximum DR of 14. The tensile strength was 484.21 ± 17.49 MPa and the Young’s modulus was 9.04 ± 0.22 GPa. While M-PP-BT-S2 reached 620.72 ± 18.46 MPa in tensile strength and 14.93 ± 0.35 GPa in Young’s modulus at DR = 17, while the maximum reached DR was 18.

The differences in mechanical properties between two DRs lie within the standard deviation. But some trends can be retrieved from the results. For all m-m fibers, the mechanical properties are improving with increased DR. Further, the compounded but unfilled PP references surpass the DR of the raw PP-100 reference. The compounding process could lead to reduced lengths of the molecular chains and resulting changed molecular weights, which influences the polymers drawability and mechanical properties, as shown by Kamezawa et al. [77]. Also, the filler-containing materials surpass the M-PP reference. But, in the case of S1, the filled M-PP-CC-S1 stays behind M-PP-S1, which can be explained by a hindered orientation of the molecular chains caused by the block-like-shaped CC particles. On the other hand, M-PP-BT-S2 surpasses all other materials clearly, which indicates an increased drawability as a result of the platelet structure of the BT filler. Van Erp et al. [78] observed a similar effect and explanation in their research on montmorillonite-filled and drawn tapes.

**Figure 5:**
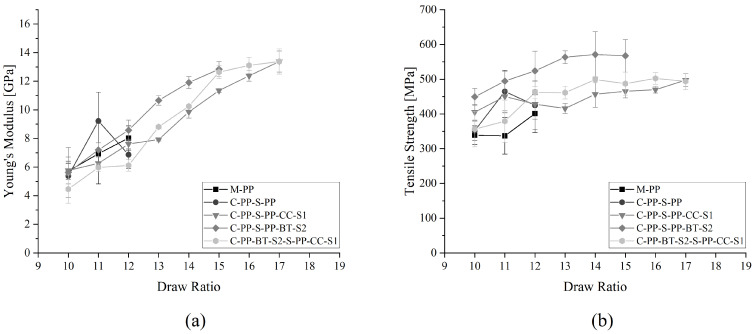
Mechanical properties of core-shell fibers at different DRs: (**a**) Young’s modulus and (**b**) tensile strength.

Figure 5 presents the mean values of Young’s modulus and tensile strength of the c-s fibers at different DRs. The c-s reference fiber C-PP-S-PP reached a maximum DR of 12, similar to M-PP. Its maximum tensile strength and Young’s modulus were 464.61 ± 61.18 MPa and 9.22 ± 2.01, respectively at DR = 11. All C-S fibers with fillers either in the shell or in the core and shell performed much better. C-PP-S-PP-CC-S1 gained a tensile strength of 497.41 ± 19.15 MPa and a Young’s modulus of 13.38 ± 0.73 GPa at its maximum DR of 17, while C-PP-S-PP-BT-S2 reached 571.01 ± 65.78 MPa as maximum tensile strength at DR = 14 and 12.84 ± 0.52 GPa in Young’s modulus at DR = 15. The fiber with fillers in the core and in the shell C-PP-BT-S2-S-PP-CC-S1 attained its highest tensile strength of 502.23 ± 17.00 MPa at DR = 16 and its maximum Young’s modulus of 13.38 ± 0.88 GPa at DR = 17.

Also for the c-s fibers, some trends and effects are visible. The fiber with BT in the shell shows a higher tensile strength compared to all other c-s fibers and reaches its maximum Young’s modulus earlier than C-PP-S-PP-CC-S1 and C-PP-BT-S2-S-PP-CC-S1, but the Young’s modulus of all three fibers is comparable. The tensile strength of C-PP-BT-S2-S-PP-CC-S1 tends to be higher than that of C-PP-S-PP-CC-S1 for most of the DRs, which indicates again the earlier mentioned possibility of an effect on the molecular orientation caused by the BT, which could be partially suppressed by the CC. On the other hand, the expected improvement in drawability and mechanical properties caused by a fiber core without CC is clearly visible.

The Young’s modulus as well as the equivalent fiber diameter and the calculated aspect ratio at the draw ratio where maximum tensile strength was reached are shown in Table 6. A comparison between the values shown in Table 5 and Table 6 can explain the variation in the mechanical properties of M-PP-S2 and C-PP-S-PP at different DRs. The equivalent fiber diameter is higher for the higher DR, which influences the mechanical properties. Usually the fiber diameter is reduced with higher DRs. M-PP-S2 and C-PP-S-PP also show high standard deviations in the mechanical properties, which indicates variations in the fiber diameter also within one DR. An uneven drawing in the furnace might be the reason for the varying fiber diameters. To understand the phenomenon completely, further research would be necessary.

To show the potential of the here presented fibers, the mechanical properties presented in Table 6 are compared with the commercially available polymer fibers for concrete reinforcements shown in Table 7. The assumed fiber length of 50 mm for the calculation of the aspect ratio lies in the middle of the commercially available fibers. However, as the fibers produced in this study have thinner diameters, they also show clearly higher aspect ratios. The mechanical properties are comparable to the commercial fibers. The Young’s modulus can be surpassed by some of the fibers with higher DRs as well as the tensile strength. Only the BarChip fibers could not be reached.

### 3.2. Density

**Figure 6:**
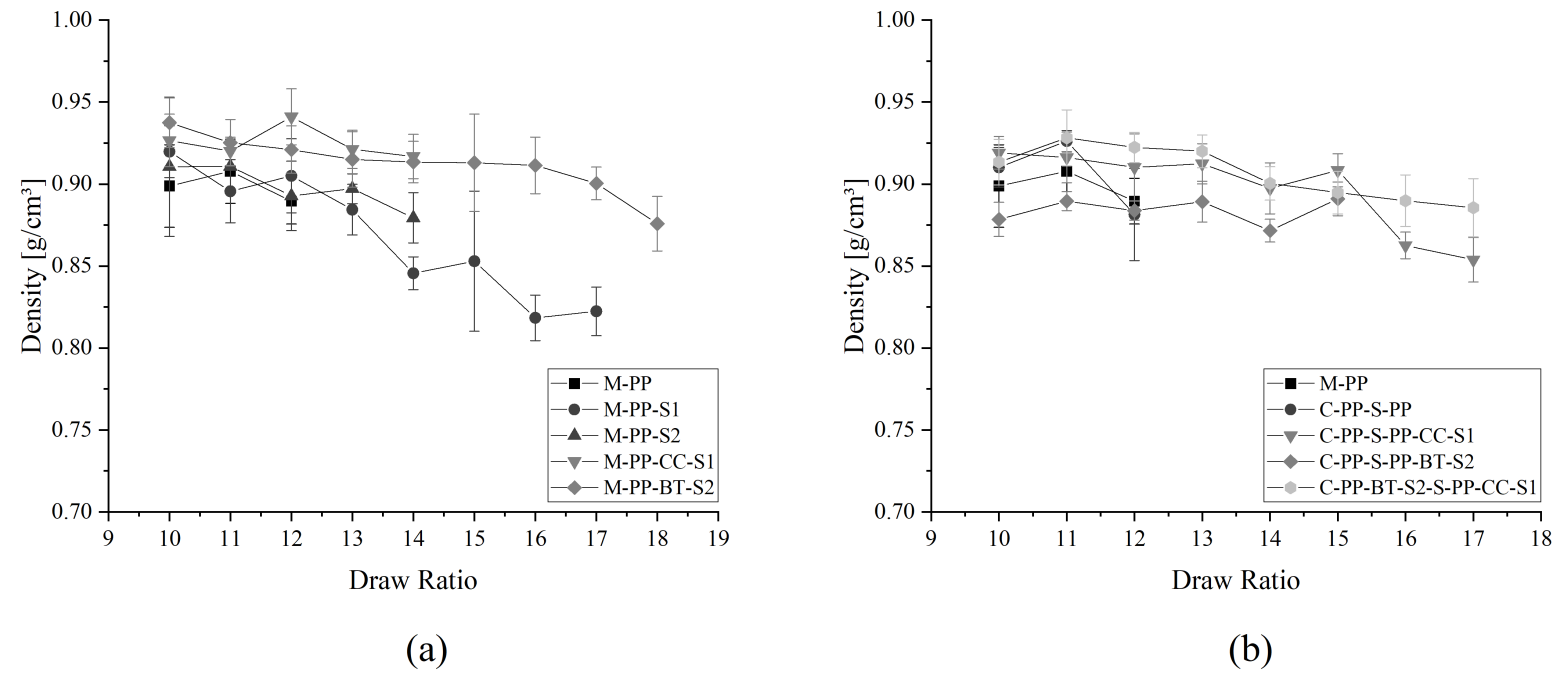
Density of different fibers at different DRs: (**a**) for mono-material fibers and (**b**) for core-shell fibers.

The symbols in Figure 6 represent the densities of the m-m and c-s fibers at their different draw ratios.

All fibers show decreasing densities with increasing draw ratio. In the m-m fibers in Figure 6a, two groups are visible. The first group, all materials without fillers, shows comparable densities and a similar development over DR. The second group, M-PP-CC-S1 and M-PP-BT-S2, shows a higher starting density, which holds on in comparison with the unfilled materials. This difference is a result of the added fillers. CC, which is based mainly on calcite, has a density of 2.72 g/cm^3^ [93], while the BT has 1.8 g/cm^3^ [66]. Accordingly, the density of the composite materials is higher. During fiber drawing, voids are created in the drawing direction around the fillers, as shown by different publications [35,94,95,96], which reduces the density of the fibers with increasing DR. The decreasing density of the fibers without fillers also results from created voids during drawing. These structural changes have been well described for polyolefins by different authors [95,97,98]. The here presented results also fit in with the observations by Baltá-Calleja and Peterlin [99] and Sokkar et al. [100], which both describe a reduction in density as a drawing effect. In terms of the c-s fibers in Figure 6b, no clear difference between the unfilled fibers and the ones containing fillers is visible. But the same density-decreasing trends for higher draw ratios as presented and explained for the m-m fibers are visible.

### 3.3. Contact Angle Measurement

**Figure 7:**
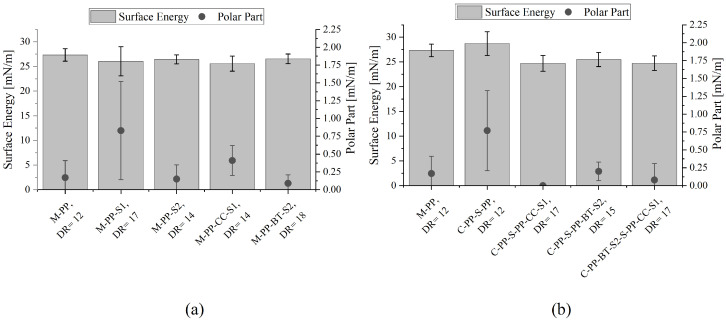
Surface energy and polar part for different fibers at their maximum MDRs: (**a**) for mono-material fibers and (**b**) for core-shell fibers.

The average surface energy and polar parts of the m-m and c-s fibers at their maximum DR are shown in Figure 7. The results yielded no signs of influence by the added fillers in the m-m fibers, as all surface energies lie in the same range between 25.57 ± 1.53 and 27.32 ± 1.25 mN/m. Also, the polarity is low with a maximum value of 0.83 ± 0.69 mN/m for M-PP-S1 (see Figure 7a). The same is shown in the case of the c-s fibers. The highest surface energy and polar part are reached by the reference c-s fiber (C-PP-S-PP) with 28.71 ± 2.4 mN/m and 0.77 ± 0.56 mN/m, respectively (see Figure 7b). It seems like there is a slight decrease in the surface energy of the filled c-s fibers compared to their m-m counterparts, but the mean values are lying in the standard deviations of each other.

### 3.4. Topography Measurement

**Figure 8:**
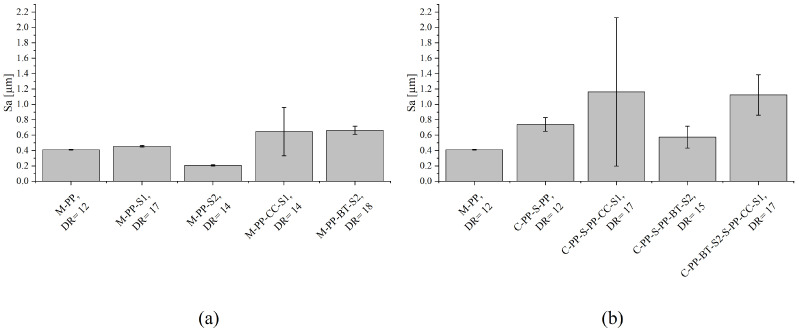
Average surface height for different fibers at their maximum MDRs: (**a**) for mono-material fibers and (**b**) for core-shell fibers.

**Figure 9:**
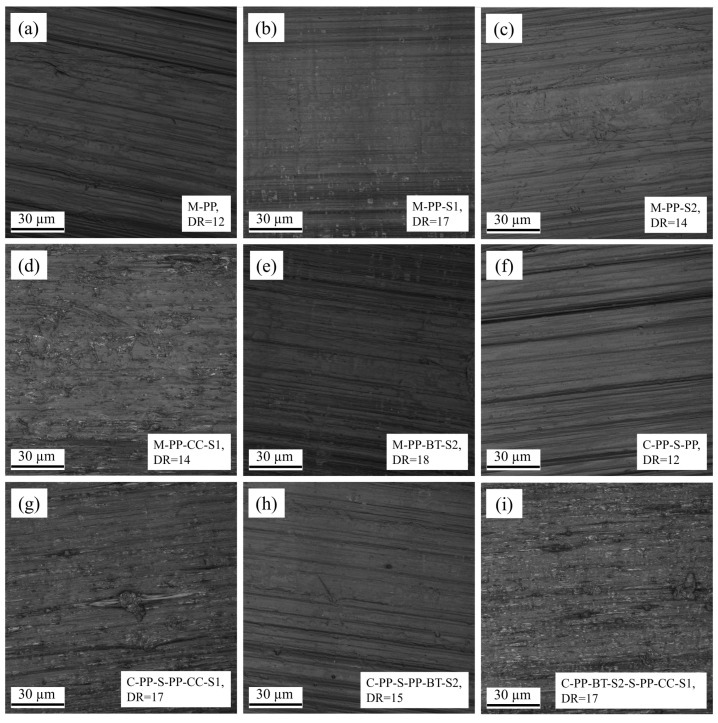
Pictures obtained from convocal laser scanning microscopy, (**a**) M-PP, (**b**) M-PP-S1, (**c**) M-PP-S2, (**d**) M-PP-CC-S1, (**e**) M-PP-BT-S2, (**f**) C-PP-S-PP, (**g**) PP-S-PP-CC-S1, (**h**) PP-S-PP-BT-S2, (**i**) C-PP-BT-S2-S-PP-CC-S1.

Figure 8 presents the average surface heights of all fibers investigated in this study at their maximum DR. Further, Figure 9 shows one representative picture obtained from CLSM for every fiber material. The m-m fibers without fillers show low surface heights between 0.21 ± 0.01 µm for M-PP-S2 and 0.46 ± 0.01 µm for M-PP-S1. These are surpassed by filled m-m fibers, where M-PP-CC-S1 reaches 0.64 ± 0.31 µm and M-PP-BT-S2 reaches 0.66 ± 0.05 µm (see Figure 8a). A comparison of Figure 9a–e matches these results, as there is a lot more texture visible for the filler containing fibers.

The average surface heights shown in Figure 8b show a higher value for the c-s reference fiber (C-PP-S-PP) compared to the m-m one (M-PP). This could be caused by the different dyes used for the production of m-m and c-s fibers. Further, there is a huge increase visible in the surface heights for the fibers containing CC in the shell compared to the reference fiber as well as the accompanying m-m fiber. This can be explained by a lot of texture that is generated by the CC particles and resulting filler particles that stick out of the surface and create voids, like it is shown in Figure 9g,i. For C-PP-S-PP-BT-S2, there is just a slight increase in the average heights visible compared to M-PP-BT-S2. This also fits with the CLSM pictures, where a nearly similar surface is visible.

### 3.5. Single-Fiber Pull-Out Test

**Figure 10:**
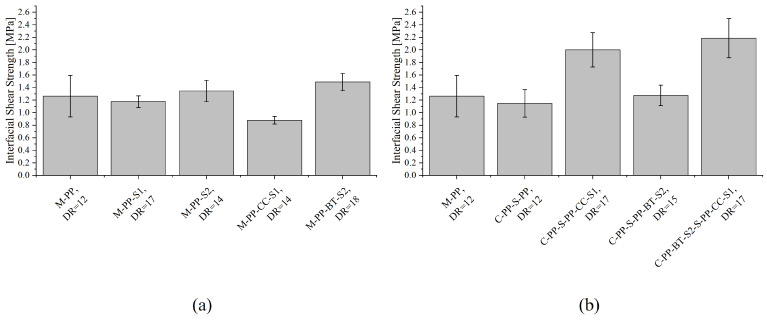
Interfacial shear strength for different fibers at their maximum MDRs: (**a**) for mono-material fibers and (**b**) for core-shell fibers.

The average IFSS retrieved from the SFPT is presented for all m-m and c-s fibers in Figure 10. The reference materials without filler (M-PP, M-PP-S1, M-PP-S2, C-PP-S-PP) show all values between 1.15 ± 0.22 MPa and 1.35 ± 0.17 MPa. M-PP-CC-S1 undercuts these values, while M-PP-BT-S2 reaches a slightly higher IFSS of 1.49 ± 0.14 MPa. The filler containing c-s fibers reach 2.0 ± 0.27 MPa (C-PP-S-PP-CC-S1) or 2.19 ± 0.31 MPa (C-PP-BT-S2-S-PP-CC-S1) clearly higher IFSS than the m-m or reference fibers, as long as CC is in the shell material. The IFSS of C-PP-S-PP-BT-S2 is 1.27 ± 0.16 MPa, just in the range of the reference materials.

Babafemi and Boshoff [44] state multiple fiber properties like fiber strength or elastic modulus influence the bonding behavior of a fiber in concrete, and Sigrüner et al. [32] add fiber roughness and polarity as supporting factors. Accordingly, to explain the resulting IFSSs, the results from the other analyses presented earlier in this study should be consulted. As the results from contact angle measurements showed no huge differences in surface energy or polarity between the different m-m and c-s fibers, an influence on their bonding behavior from this side is not supported within this study. But the average surface heights correlate for some materials very well with the observed IFSSs. The highest IFSS was reached by the c-s fibers containing CC in the shell, which are the same fibers that achieved the highest Sa values. On the other hand, the Sa values for the two filled m-m fibers are nearly similar, but the IFSS of M-PP-BT-S2 is clearly higher than the one of M-PP-CC-S1. An explanation for this effect could be the huge difference in the mechanical properties, where M-PP-BT-S2 performs much better. More difficult is the explanation of the similar IFSS for all materials without fillers, as they showed differences in their Sa values as well as their mechanical properties. Possibly, these differences observed in mechanical properties and surface topography are not high enough. A comparison between the m-m and the c-s fiber with BT particles in the fiber or shell material fits again the idea of a supporting influence of mechanical properties and surface topography on the IFSS, as all these values are slightly higher for the m-m fibers.

### 3.6. Reflected Light Microscopy

**Figure 11:**
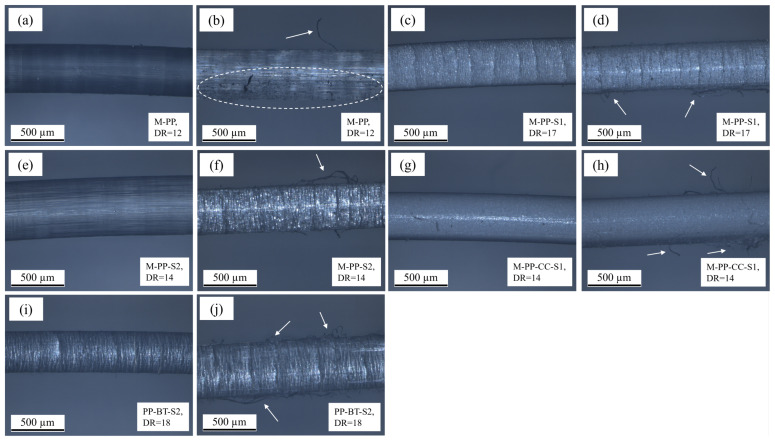
Pictures obtained from reflected light microscopy on m-m fibers, (**a**) M-PP before pull-out, (**b**) M-PP after pull-out with slightly changed surface, adhering concrete and few fibrils, (**c**) M-PP-S1 before pull-out, (**d**) M-PP-S1 after pull-out with some fibrils, (**e**) M-PP-S2 before pull-out, (**f**) M-PP-S2 after pull-out with highly changed surface and few fibrils, (**g**) M-PP-CC-S1 before pull-out, (**h**) M-PP-CC-S1 after pull-out with few fibrils, (**i**) M-PP-BT-S2 before pull-out, (**j**) M-PP-BT-S2 after pull out with fibrils. The arrows and the dashed ellipse mark the mentioned deformations.

**Figure 12:**
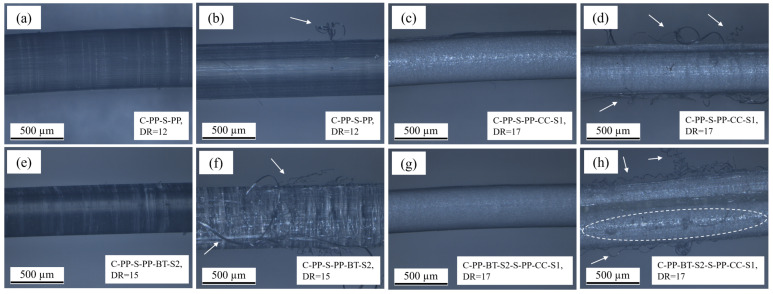
Pictures obtained from reflected light microscopy on c-s fibers, (**a**) C-PP-S-PP before pull-out, (**b**) PP-S-PP after pull-out with slightly changed surface and very few fibrils, (**c**) PP-S-PP-CC-S1 before pull-out, (**d**) PP-S-PP-CC-S1 after pull-out with fibrils, (**e**) PP-S-PP-BT–S2 before pull out, (**f**) PP-S-PP-BT-S2 after pull-out with highly chaged surface and fibrils, (**g**) C-PP-BT-S2-S-PP-CC-S1 before pull-out, (**h**) C-PP-BT-S2-S-PP-CC-S1 after pull-out with changed surface, adhering concrete and fibrils. The arrows and the dashed ellipse mark the mentioned deformations.

The pictures recorded on fibers at their maximum draw ratio before and after pull-out by RLM Figure are shown for m-m fibers in Figure 11 and for c-s fibers in Figure 12. All fibers show a smooth and regular surface with some visible differences before pull-out. In a comparison between the M-PP fiber (Figure 11a) and the M-PP-S1 (Figure 11c), there are different structures discernible, which result from the different DRs. Also, the fillers in the m-m fibers cause different structures (see Figure 11g,i). It stands to reason that the pictures obtained from RLM show not only the surface but also some reflecting structures below the surface, as the circulating markings on the fibers in Figure 12a,e. The fibers containing CC show quite similar surfaces whether they are m-m or c-s fibers (see Figure 11g and Figure 12c,g), while there are slight differences in the other cases.

After pull-out, multiple changes to the m-m and c-s fibers can be observed. The M-PP fiber shows a changed surface with adhering concrete and few fibrils (see Figure 11b), while on C-PP-S-PP, which has the same DR, also few fibrils but no adhering concrete are visible (see Figure 12b). The other fibers without filler indicate an influence of the DR on the deformation during pull-out. M-PP-S2 at DR = 14 shows a highly worn-out surface with fibrils (see Figure 11f), which is again lower than DR = 17 for M-PP-S1, where only some surface damage and fibrils are visible (see Figure 11d). The different damage mechanisms might be an explanation for the similar pull-out results, although the results in surface topography and mechanical properties indicated a different trend.

In the case of M-PP-CC-S1, some fibrils are visible after pull-out, but the surface appears unchanged (see Figure 11h). For C-PP-S-PP-CC-S1 (Figure 12d), there are more and longer fibrils visible compared to the m-m fiber with CC. Also in the picture from C-PP-BT-S2-S-PP-CC-S1 after pullout (Figure 12h), more fibrils and a deformed surface with some adhering concrete are visible. These higher surface deformations fit the higher IFSS reached by the c-s fibers.

In Figure 11j, multiple fibrils are discernible, created by the pull-out of an M-PP-BT-S2 fiber. Compared to that, the C-PP-S-PP-BT-S2 shows the same deformation with even more fibrils (see Figure 12f). As there are just slight differences in IFSS as well as mechanical properties and Sa, only small differences in the observable deformation seem to be logical.

### 3.7. Thermograviemtric Analysis

**Figure 13:**
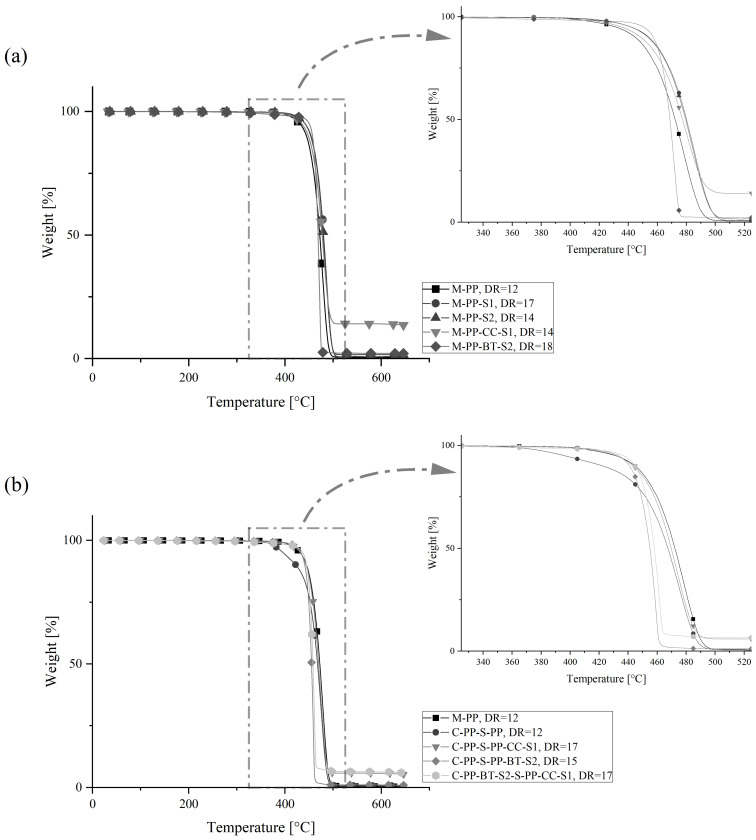
TGA results: weight loss over temperature with enlarged temperature scales for the area of combustion for (**a**) m-m fibers and (**b**) for c-s fibers.

The results from thermogravimetric analysis are shown in Table 8 by different comparative parameters like the onset point, the temperature at different weight losses, and the maximum degradation rate derived from the TGA curves and Figure 13, which provides an overview of the complete temperature range and an enlarged view on the degradation section of the m-m and c-s fibers at their maximum DRs.

In general, there are no huge differences visible for all the m-m and c-s fibers. But some smaller effects can be discussed. The M-PP fiber degrades slightly earlier than M-PP-S1 and M-PP–S2, which is also indicated by a difference in the onset temperature of ≈12 °C. This could be a result of the additional processing in the compounding process, where early degrading volatiles might be removed by the vacuum degassing system used. The degradation curve of M-PP-CC-S1 lies between the ones from unfilled PP, which indicates no change in the thermal degradation behavior by the CC, but the fiber shows the expected residue at the end temperature. The m-m fiber containing BT shows a different thermal degradation compared to the other m-m fibers, with a higher onset temperature and later a higher degradation rate. This corresponds to the use of BT as a flame retardant in polyolefins shown in the literature by multiple authors [101,102,103,104].

The c-s fibers show similar behavior. The C-PP-S-PP fiber degrades slightly earlier compared to the M-PP fiber, which might be caused by an enhanced active surface during TGA as a result of delaminated c-s layers during sample preparation. The fillers cause similar effects in the core-shell fibers like in the m-m fibers. There is again a combustion residue visible if CC is in the fiber shell, and a slightly delayed degradation onset with a later increased degradation rate can be observed if BT is in the fiber core or shell.

In concrete applications, the combustion of PP fibers during a fire can reduce spalling, as this creates a porous structure that allows evaporating water to escape [105]. Accordingly, the fibers containing BT could be the best solution here, as they show, on the one hand, a higher onset temperature and are completely degraded earlier on the other hand.

## 4. Conclusions

M-m and c-s fibers were produced using raw PP and compounds with CC and BT by compounding the filled materials, extrusion and coextrusion of the strands, and drawing of the strands to fibers. The produced fibers were investigated for their suitability as concrete reinforcement. The tensile tests show an increase in the mechanical properties with increasing DR. The addition of CC in m-m fibers lowered the reachable mechanical properties, while the addition of BT led to a huge increase. In C-S fibers, the negative effect of CC on the mechanical properties could be negotiated by the used core components. The density of all materials decreased with increasing DR. The contact angle measurements indicated no change by the added fillers in m-m as well as c-s fibers. But the surface topography measurements showed a high average surface height for c-s fibers containing CC in the shell. Also, the IFSS retrieved from SFPTs was highest for the c-s fibers with the CC compound in the shell, which reached ≈1.5 times higher values than the unfilled references. Pictures recorded by RLM show different fiber surfaces before pull-out and changed surface structures, fibrils, and adhering concrete after pull-out. The TGA makes a changed thermal degradation visible for fibers containing BT.

This study also gives an insight into the possibilities of c-s fibers made from PP with fillers either in the shell or in the core and the shell. The optimum fiber is made with a core made from PP and BT and a shell of PP and CC, which leads to acceptable mechanical properties with an improved anchoring of the fiber in concrete. Further, the thermal decomposition is favorable.

Nevertheless, further research would be necessary to improve the fiber properties on the one hand and to prove the reinforcement potential of the fiber on the other. Next steps could be an investigation with other fillers with similar structure, cubic-shaped particles in the shell and platelet-shaped particles in the core, as well as different filler loadings. The reinforcement potential could be evaluated using more realistic test setups, like a compressive or three-point bending test on fiber-reinforced concrete specimens with different filler loadings. 

## Figures and Tables

**Table 1:** Concrete mixture.

Material		Sand	Portland Cement	Limestone Powder	Water	Superplasticizer	
						1	2
Amount	[g]	1350	450	75	220	6.2	3.0
	[wt.%]	64.15	21.38	3.56	10.45	0.29	0.15

**Table 2:** Denotations and formulations of used fiber materials.

Material Denotations	Amount of PP	Amount of Filler		Amount of MAH	Screw Configuration
		CC	BT		
	[wt.%]	[wt.%]		[wt.%]	[-]
PP-S1	100	-	-	-	S1
PP-CC-S1	79.58	13.61	-	6.81	S1
PP-S2	100	-	-	-	S2
PP-BT-S2	95.15	-	4.85	-	S2

**Table 3:** Parameters of the m-m strand extrusion.

Material	Extruder			Melt Pump		
	Screw Speed [rpm]	Mass Temperature [°C]	Pressure [bar]	Throughput [kg/h]	Mass Temperature [°C]	Pressure [bar]
M-PP	44	199	47	1.5	209	45
M-PP-S1	50	199	48	1.5	209	44
M-PP-CC-S1	38	198	47	1.5	205	41
M-PP-S2	65	200	48	1.5	210	45
M-PP-BT-S2	53	197	48	1.5	209	43

**Table 4:** Parameters of the c-s strand coextrusion.

Material	Extruder 1				Extruder 2		
	Screw Speed	Throughput	Mass Temperature	Pressure	Screw Speed	Mass Temperature	Pressure
	[rpm]	[kg/h]	[°C]	[bar]	[rpm]	[°C]	[bar]
C-PP-S-PP	120	1.5	217	121	20	207	124
C-PP-S-PP-CC-S1	120	1.5	218	99	20	210	116
C-PP-S-PP-BT-S2	120	1.5	218	99	20	212	110
C-PP-BT-S2-S-PP-CC-S1	120	1.5	217	90	20	211	110

**Table 5:** Properties of the produced fibers at their maximum draw ratio.

Material	Draw Ratio	Equivalent Fiber Diameter	Aspect Ratio ^1^	Young’s Modulus	Tensile Strength
	[1]	[mm]	[1]	[GPa]	[MPa]
M-PP	12	0.58 ± 0.02	85.9	8.03 ± 0.85	401.10 ± 54.31
M-PP-S1	17	0.51 ± 0.01	97.5	12.74 ± 0.52	475.04 ± 19.32
M-PP-S2	14	0.57 ± 0.11	87.3	10.17 ± 4.60	434.52 ± 139.10
M-PP-CC-S1	14	0.49 ± 0.00	102.7	9.04 ± 0.22	484.21 ± 17.49
M-PP-BT-S2	18	0.46 ± 0.02	108.4	13.88 ± 1.28	613.40 ± 12.88
C-PP-S-PP	12	0.60 ± 0.04	83.1	6.87 ± 0.96	424.77 ± 69.84
C-PP-S-PP-CC-S1	17	0.49 ± 0.02	101.9	13.38 ± 0.73	497.41 ± 19.15
C-PP-S-PP-BT-S2	15	0.49 ± 0.01	101.4	12.84 ± 0.52	567.42 ± 46.73
C-PP-BT-S2-S-PP-CC-S1	17	0.50 ± 0.01	100.4	13.38 ± 0.88	493.71 ± 23.50

^1^ Calculated with an assumed fiber length of 50 mm.

**Table 6:** Properties of the produced fibers when they reach their maximum tensile strength.

Material	Draw Ratio	Equivalent Fiber Diameter	Aspect Ratio ^1^	Young’s Modulus	Tensile Strength
	[1]	[mm]	[1]	[GPa]	[MPa]
M-PP	12	0.58 ± 0.02	85.9	8.03 ± 0.85	401.10 ± 54.31
M-PP-S1	16	0.50 ± 0.01	100.5	13.23 ± 0.60	514.88 ± 21.65
M-PP-S2	13	0.52 ± 0.01	96.4	11.94 ± 1.04	491.72 ± 38.68
M-PP-CC-S1	14	0.49 ± 0.00	102.7	9.04 ± 0.22	484.21 ± 17.49
M-PP-BT-S2	17	0.47 ± 0.01	107.1	14.94 ± 0.35	620.72 ± 18.46
C-PP-S-PP	11	0.54 ± 0.04	92.3	9.22 ± 2.01	464.61 ± 61.18
C-PP-S-PP-CC-S1	17	0.49 ± 0.02	101.9	13.38 ± 0.73	497.41 ± 19.15
C-PP-S-PP-BT-S2	14	0.50 ± 0.02	99.3	11.91 ± 0.41	571.01 ± 65.78
C-PP-BT-S2-S-PP-CC-S1	16	0.51 ± 0.00	98.3	13.11 ± 0.55	502.24 ± 17.00

^1^ Calculated with an assumed fiber length of 50 mm.

**Table 7:** Properties of different commerically available fibers.

Manufacturer	Fiber Name	Equivalent Fiber Diameter	Length	Aspect Ratio	Young’s Modulus	Tenisle Strength	Source
		[mm]	[mm]	[-]	[GPa]	[MPa]	
Advil	Durus^®^ S500 48 mm	0.7	48	69	6	500	[79]
	Durus^®^ EasyFinish	0.7	40	57	6	500	[80]
BarChip Inc.	BarChip 48	- ^1^	48	- ^1^	12	640	[81]
	BarChip 60	- ^1^	60	- ^1^	12	640	[82]
Contec Fiber AG	Concrix M507	0.75	50	67 ^2^	6	450	[83]
	Concrix SA	0.5	25	50 ^2^	11	620	[84]
Kordsa Teknik Tekstil A.Ş.	Kratos Macro PP 48	0.72	48	67	8.5	550	[85]
	Kratos Macro PP 54	0.72	57	75	8.5	550	[86]
MAPEI^®^	MAPEFIBRE ST 42	0.8	42	53 ^2^	3.9	450	[87]
	MAPEFIBRE ST30	0.8	30	38 ^2^	3.8	450	[88]
Master Builders Solutions	Master Fiber 255 SPA	0.7	55	79	>8	500	[89]
	MasterFiber^®^ 245 SPA	0.7	48	69	≥8	500	[90]
Sika Deutschland GmbH	SikaFiber^®^ Force-50	≈0.73	≈50	68 ^2^	7.5	450	[91]
	SikaFiber^®^-40 Force	≈0.75	≈40	53 ^2^	8	480	[92]

^1^ Not provided by the manufacturer. ^2^ Calculated from the provided equivalent fiber diameter and fiber length.

**Table 8:** Resulst form TGA.

Material	Draw Ratio [-]	Onset Point [°C]	Tempreature at			Maximum
			1% Weight Loss [°C]	2% Weight Loss [°C]	5% Weight Loss [°C]	Degradation Rate [%/°C]
M-PP	12	454.71	398.25	411.07	431.41	−3.24
M-PP-S1	17	463.60	411.27	424.17	442.00	−3.65
M-PP-S2	14	462.60	408.71	423.25	441.87	−3.28
M-PP-CC-S1	14	457.07	401.33	416.84	435.23	−2.77
M-PP-BT-S2	18	462.57	359.75	419.10	450.59	−8.06
C-PP-S-PP	12	448.49	363.56	375.51	396.21	−2.73
C-PP-S-PP-95-CC-S1	17	485.39	403.05	414.96	431.86	−3.11
C-PP-S-PP-BT-S2	15	449.85	398.37	417.80	433.27	−9.12
C-PP-BT-S2-S-PP-CC-S1	17	451.88	363.71	413.37	436.91	−7.98

## Data Availability

The original contributions presented in this study are included in the article. Further inquiries can be directed to the corresponding authors.

## Abbreviations

The following abbreviations are used in this manuscript:

BT	Bentonite
C	Core
c-s	core-shell
CLSM	Convocal Laser Scanning Microskopy
DR	Draw Ratio
DSC	Differential Scanning Calorimetry
IFSS	Interfacial shear strength
m-m	mono-material
PP	Polypropylene
S	Shell
SFPT	Single Fiber Pull-out Test
TGA	Thermogravimetric Analysis
vol.%	Volume Percent
wt.%	Weight Percent
